# Hemoperitoneum due to aneurysm rupture related to systemic lupus erythematosus: a case report

**DOI:** 10.1093/jscr/rjaf256

**Published:** 2025-04-28

**Authors:** Ryoma Sakamoto, Yuto Kawate, Satomi Kono, Satoshi Matsuda, Hiroshi Kusanagi

**Affiliations:** Department of Gastrointestinal Surgery, Kameda Medical Center, 929, Higashimachi, Kamogawa, Chiba 2960041, Japan; Department of Gastrointestinal Surgery, Kameda Medical Center, 929, Higashimachi, Kamogawa, Chiba 2960041, Japan; Department of Gastrointestinal Surgery, Kameda Medical Center, 929, Higashimachi, Kamogawa, Chiba 2960041, Japan; Department of Gastrointestinal Surgery, Kameda Medical Center, 929, Higashimachi, Kamogawa, Chiba 2960041, Japan; Department of Gastrointestinal Surgery, Kameda Medical Center, 929, Higashimachi, Kamogawa, Chiba 2960041, Japan

**Keywords:** SLE, aneurysm, rupture

## Abstract

Although systemic lupus erythematosus (SLE) is often associated with aneurysm formation, hemoperitoneum due to SLE-related abdominal visceral aneurysm is rare. A 53-year-old man with a 21-year history of SLE presented to our hospital with abdominal pain. Contrast-enhanced computed tomography revealed extravasation around a branch artery of the inferior mesenteric artery (IMA), suggesting hemorrhage from the same region. Emergency laparotomy was performed due to patient’s declining condition. A large hematoma in the mesentery of the splenic flexure of the descending colon was observed, and resection of the descending colon and hematoma was performed. Pathological examination revealed aneurysmal dilation of an IMA branch within the hematoma. Affected blood vessels exhibited extreme thinning due to fibrinoid necrosis, possibly caused by SLE. The patient was discharged 28 days after the initial surgery without complications. Aneurysm rupture should be considered in the differential diagnosis of acute abdomen in patients with SLE.

## Introduction

Systemic lupus erythematosus (SLE) is often associated with aneurysms; however, hemoperitoneum due to SLE-associated rupture of abdominal visceral aneurysms is extremely rare, with very few similar reports. Herein, we describe a case of hemoperitoneum caused by spontaneous rupture of an SLE-related aneurysm, in which a two-stage operation was performed and the patient survived.

## Case report

**Figure 1:**
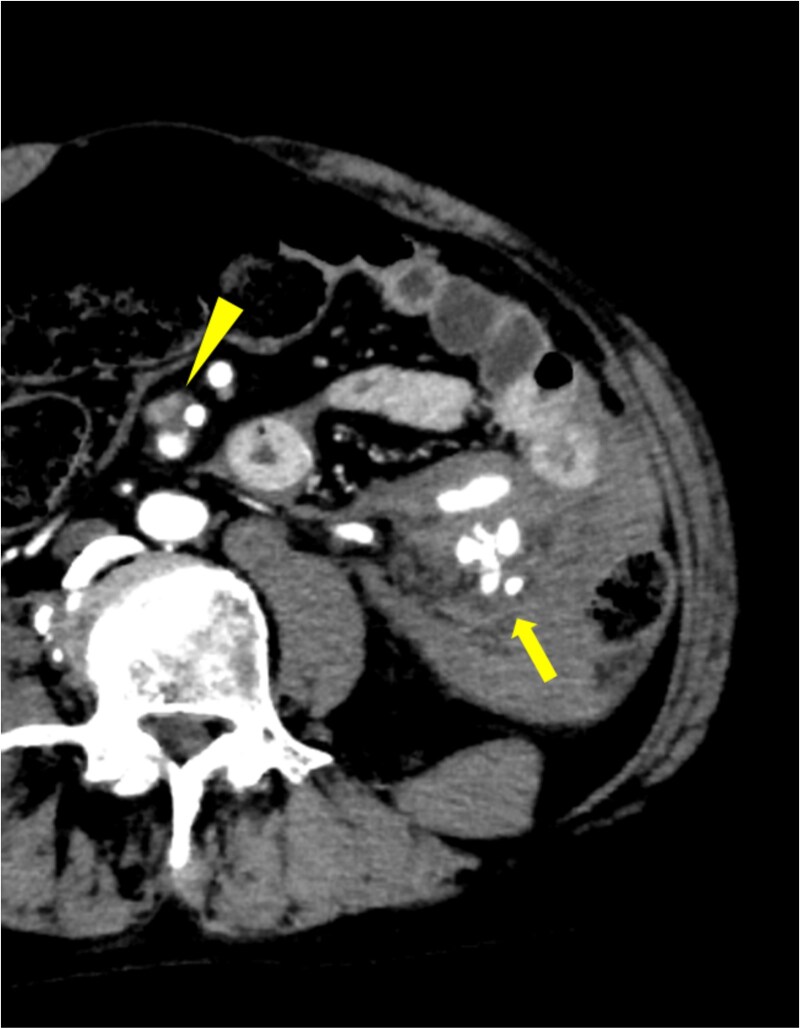
Extravasation continuous from IMA was observed (solid arrow), and active bleeding from the same region was suspected. Aneurysm with internal thrombus was observed near the origin of SMA (arrowhead).

**Figure 2:**
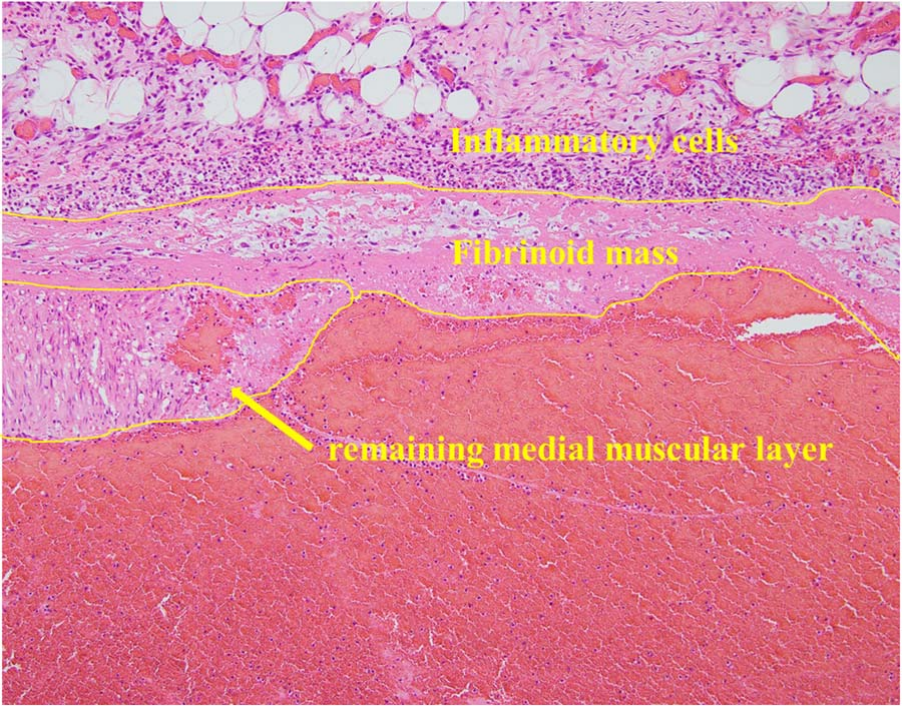
The vessel wall of the dilated artery was completely necrotic, and only a fibrin layer remained. In addition, an inflammatory cell infiltrate consisting primarily of neutrophils was observed around the affected blood vessel.

A 53-year-old man with a 21-year history of SLE presented to our emergency department with sudden onset of abdominal pain during defecation. His past medical history included SLE, deep venous thrombosis (DVT), refractory symptomatic epilepsy, and neurogenic bladder. His medications included predonine (used for 21 years), warfarin (for DVT), and antiepileptic drugs. His blood pressure was 100/60 mmHg, and his heart rate was 140 bpm. Physical examination revealed abdominal distention, strong spontaneous pain, and tenderness in the left side of the abdomen. Furthermore, the patient had rebound tenderness in the same region. Laboratory examination revealed severe anemia (Hb 8.4 g/dL) and elevated inflammatory markers (C-reactive protein: 7.90 mg/dL). Both prothrombin time (PT) and activated partial thromboplastin time (APTT) were prolonged (PT: 57.8 s, APTT: 71.2 s). Contrast-enhanced computed tomography (CT) of the abdomen revealed an extravasation in the left side of the abdomen, continuous from the inferior mesenteric artery (IMA). In addition, a spindle-shaped dilatation with an internal contrast-deficient area with a maximum diameter of 17 mm, was observed 16 mm peripherally from the origin of the superior mesenteric artery (SMA) (Fig. 1). No areas of contrast failure were observed in the intestinal tract. Emergency laparotomy was performed on the same day due to patient’s declining condition. A large amount of bloody ascites and clots were present in the abdominal cavity. A hematoma, ⁓10 cm in size was found in the mesentery of the splenic flexure of the colon and was considered the source of hemorrhage. No other sources of hemorrhage were found. Hemostasis was achieved through compression. Although we considered closing the abdomen and performing interventional radiology for hemostasis, there was a risk of ischemic necrosis of the descending colon after embolization. Therefore, we decided to excise the descending colon, including the hematoma. The intestinal tract and mesentery from the splenic flexure to the middle of the descending colon were resected to remove all the hematoma. Due to significant intestinal edema caused by aggressive hydration, we could not close the abdomen and opted for open abdominal management without intestinal anastomosis. A second look surgery was performed 2 days after the initial surgery. The risk of anastomotic leak was considered high based on the patient’s condition, so descending colostomy was performed. The patient was discharged 28 days after the initial surgery without any postoperative complications. The resected descending colon had a large, bulging hematoma on the serosal side, measuring 9 × 6 cm. Histopathological examination of the resected colon revealed mild but diffuse dilatation of a major branch of the IMA running parallel to the bowel, which was embedded within the hematoma. The structure of the dilated artery was completely destroyed, leaving only the fibrin layer. Inflammatory infiltrate, including neutrophils, was observed around the affected vessels (Fig. 2). The bowel wall was intact. Other than SLE, the patient had no comorbidities that could cause the formation of multiple aneurysms in the SMA and IMA. Therefore, this aneurysm was suspected to be caused by SLE.

## Discussion

Although aneurysms are sometimes observed in patients with SLE [1, 2], the relationship between aneurysms and SLE has not been fully elucidated. Inflammation of blood vessels and weakening of blood vessel walls due to long-term steroid use have been reported as the primary mechanisms of aneurysm formation [3]. Additionally, SLE is associated with accelerated atherosclerosis risk, increasing the risk of cardiovascular events [4]. In this case, arterial wall thinning due to inflammation was observed, which may have contributed to the aneurysm formation. Hemoperitoneum due to SLE-related aneurysm rupture is extremely rare. To our knowledge, only two similar cases have been reported so far [5, 6]. Across these three cases including our case, no specific trend was observed in the site of aneurysm formation, and the history of steroid use varied from 2 to 21 years. In our case, the aneurysm rupture and hemoperitoneum were probably caused by a combination of factors, such as long-term steroid use, anticoagulant use, and thinning of the vessel wall due to SLE. Aneurysm rupture is a rare but potentially fatal event; CT surveillance may be beneficial in patients with autoimmune diseases such as SLE.
